# A novel approach for the EEG-driven assessment of divided attention through mutual information theory: A case study at the wheel

**DOI:** 10.1371/journal.pone.0348608

**Published:** 2026-05-26

**Authors:** Vincenzo Ronca, Rossella Capotorto, Andrea Giorgi, Francesca Dello Iacono, Marianna Cecchetti, Linda Napoletano, Francois Brambati, Gianluca Borghini, Pietro Aricò, Gianluca Di Flumeri

**Affiliations:** 1 Department of Computer, Control, and Management Engineering “Antonio Ruberti”, Sapienza University of Rome, Rome, Italy; 2 BrainSigns srl, Rome, Italy; 3 Department of Anatomical, Histological, Forensic & Orthopaedic Sciences, Sapienza University of Rome, Rome, Italy; 4 DeepBlue srl, Rome, Italy; 5 Department of Molecular Medicine, Sapienza University of Rome, Rome, Italy; Drexel University School of Biomedical Engineering Science and Health Systems, UNITED STATES OF AMERICA

## Abstract

Monitoring how attention is distributed across concurrent task demands is fundamental in daily life contexts such as mobility, education and industrial control. However, existing neurophysiological measures, typically based on univariate EEG markers, are sensitive to generic cognitive load, visual complexity, or motor activity, and therefore lack specificity for attentional splitting. Here, we introduce the Attentional Split Index (ASI), a novel EEG-based metric that employ Mutual Information (MI) theory, designed to quantify the coordinated modulation of multiple neurometrics that emerges when attention is divided across tasks. Twenty-five participants completed a realistic driving protocol combining a main driving task in two different environments (Urban, Highway) with four types of attentional-split demands, i.e., Focused, Auditory Continuous Performance Test (ACPT), Matrix, Surrogate Reference Task (SURT). Traditional neurometrics (parietal alpha, inverse frontal beta, frontal theta/beta ratio) exhibited partial sensitivity to task demands but failed to selectively reflect attentional splitting. In contrast, the ASI showed a robust and systematic increase across conditions, distinguishing not only explicit multitasking segments but also subtler differences between Urban and Highway focused driving. Eye-tracking and subjective distraction rating showed a strong and significant correlation with the ASI (Urban: r_ET_ = 0.515 and r_SUB_ = 0.744; Highway: r_ET_ = 0.357 and r_SUB_ = 0.673; all p < 10^−2^), confirming high behavioural and phenomenological coherence. Surrogate analyses demonstrated that ASI effects were absent when temporal coordination across neurometrics was artificially disrupted, and that real ASI exceeded surrogate values in the vast majority of participants during multitasking but not during eyes-open baseline. Together, these findings establish the ASI as a specific, robust, and ecologically coherent neural marker of attentional splitting, with promising implications for neuroergonomics and next-generation adaptive human–machine systems.

## 1. Introduction

### 1.1. Divided Attention: theoretical background and relevance in everyday and operational contexts

Human attention is inherently limited and must be continuously distributed across multiple sources of information. This ability, commonly referred to as divided attention or attentional split, plays a fundamental role not only in applied operational domains such as driving, piloting, industrial monitoring, and surgical performance, but also in everyday activities including navigating complex environments, interacting with digital interfaces, or managing simultaneous cognitive streams [1,2]. Classical models conceptualize attention as a capacity-limited resource that must be allocated across competing demands [1]. Dual-task paradigms consistently show that simultaneous task execution leads to slower response times, increased errors, and reduced situational awareness [3,4], thus highlighting the presence of a capacity limit even if not measurable.

Beyond its theoretical relevance, the ability to measure attentional splitting carries increasing practical importance. In the automotive domain, identifying when a driver’s attention becomes divided is critical for safe human–vehicle interaction and for designing in-vehicle interfaces that minimize cognitive interference [5]. Similarly, in human–machine systems and interactive technologies, quantifying attentional split enables evaluating whether an interface imposes unintended distractions, detecting when an operator is performing an unplanned dual task, or assessing whether a user is cognitively capable of managing concurrent demands. Moreover, the ability to track attentional splitting continuously over time, rather than only across experimental conditions, is essential for real-world applications such as adaptive driver-monitoring systems, learning platforms, and human-robot collaboration.

Despite this relevance, a fundamental open question remains: is it possible to measure the extent to which a user’s attention is focused or rather divided? Experimental studies typically compare multiple controlled conditions, but real-world use requires an index that can be ‘modulated’ by how much a user’s attention is focused on a single task or divided between different cognitive demands, allowing for a robust, objective, and data-driven approach to recognising eventual risk conditions. Currently, no validated neurophysiological marker exists that can reliably indicate, moment by moment, whether attention is focal or distributed.

This gap motivates the search for a principled, continuous, and task-general index of attentional splitting.

### 1.2. State of the art in measuring Attentional Split: behavioural, oculomotor, and neurophysiological approaches

Quantifying how attention is distributed across concurrent stimuli has been the focus of extensive methodological research across cognitive psychology, human factors, and applied neuroscience. Behavioural metrics have traditionally served as the primary approach to infer attentional splitting. In dual-task and multitasking paradigms, performance decrements, such as increased reaction times, reduced accuracy, or deterioration in primary-task performance, are consistently observed as indicators of divided attention [3,4,6]. In the driving domain, secondary visual or cognitive tasks impair lane keeping, hazard detection, and braking responses [7], whereas increased task complexity during tool use or human–computer interaction similarly disrupts performance patterns [7]. Although robust, behavioural measures remain indirect indicators, often influenced by compensatory strategies, practice effects, or the prioritization of one task over another, making it difficult to attribute performance changes uniquely to attentional splitting.

Oculomotor measures offer richer temporal resolution and have proven particularly informative for assessing attentional allocation in both laboratory and naturalistic contexts. Eye-tracking studies show that divided attention increases gaze entropy, saccadic frequency, and gaze dispersion, particularly when the secondary task competes with the primary task’s visual demands [8,9]. In driving research, off-road glances reliably indicate attentional capture by secondary interfaces or in-vehicle systems [10]. In naturalistic behaviour, mobile eye-tracking has shown that multitasking alters visual search strategies and scanning efficiency [11]. Pupillary responses also correlate with cognitive load and distraction [12,13], but they are sensitive to illumination changes and emotional arousal, limiting their specificity. Thus, although behavioural and oculomotor measures are valuable for monitoring attentional allocation, they remain influenced by multiple cognitive or environmental factors and cannot, alone, isolate the neural mechanisms underlying attentional splitting.

These considerations have increasingly motivated the use of neurophysiological approaches, particularly electroencephalography (EEG). EEG offers high-level temporal resolution, allowing detection of rapid fluctuations in attentional allocation, and recent technological developments have enabled its use in ecological or semi-ecological environments such as real-world and realistic driving [14–17], outdoor walking [18], workplace monitoring, and aviation [19]. Wearable EEG systems now allow cognitive monitoring with minimal interference, making EEG an ideal candidate for detecting mental impairments and phenomena, such as the attentional splitting itself, paving the way for new and enhanced Human-Machine Interaction solutions [20–22].

Several EEG features have been consistently associated with different “attention modalities” across a wide range of experimental and ecological paradigms. In dual-task driving studies, increases in frontal theta and decreases in posterior alpha have been repeatedly observed when drivers concurrently solve cognitive tasks or respond to visual distractors [23,24]. Multitasking during locomotion, such as walking while performing mental arithmetic, has been shown to reduce P300 amplitude and modulate alpha and beta rhythms, reflecting the reallocation of attentional resources under concurrent cognitive–motor demands [18]. Complementary evidence shows that divided attention alters movement-related cortical potentials (MRCPs), with reductions in MRCP amplitude during dual-task execution indicating reduced availability of neural resources for motor preparation [25].

Beyond sensorimotor tasks, EEG modulations during problem-solving tasks requiring attention to multiple informational streams have highlighted changes in frontal theta and parietal alpha [26]. In cross-modal paradigms, divided attention reduces cross-modal congruence effects and modulates midline theta and posterior alpha, indicating distributed reconfiguration of attentional networks [27]. Neuroimaging studies further show that divided attention engages anterior cingulate, dorsolateral prefrontal, and parietal regions, often detectable in EEG through oscillatory power modulations or event-related potentials in the corresponding scalp sites [28].

Within this literature, specific neurometrics, i.e., intended as the combination of EEG spectral features, have emerged as the most commonly used to characterise attentional demand: the frontal theta/beta ratio, parietal alpha suppression, and inverse frontal beta activity. The frontal theta/beta ratio was originally introduced within ADHD research as an index of executive control [29] and has since been linked to attentional stability, task engagement and susceptibility to distraction in demanding environments [30,31]. Parietal alpha suppression, i.e., a reduction of alpha-band power over posterior regions, has long been associated with shifts in visuospatial attention, top-down allocation of cognitive resources, and the necessity to process multiple information sources simultaneously [32,33]. In parallel, inverse frontal beta activity, typically expressed as reduced beta-band power over frontal regions, has been associated with diminished top-down cognitive control and greater distractibility, particularly in sustained attention or dual-task paradigms [34,35]. Other neurometric markers have also been explored, such as beta desynchronization during task switching or divided attention [36], alpha–theta interactions associated with mental workload [37,38], and GFP-based indices reflecting high levels of engagement, workload and vigilance in operational tasks [39–42]. Collectively, these neurometrics provide physiologically interpretable windows into specific aspects of attentional processing: frontal rhythms reflecting executive control and cognitive effort, parietal rhythms indexing sensory and attentional allocation, and frontal beta dynamics reflecting top-down control.

However, while each neurometric reliably responds to manipulations involving attention and distraction, none of them is specific to the phenomenon of attentional splitting. The frontal theta/beta ratio, for example, is strongly modulated by mental effort, executive control, and inhibitory demands even in the absence of divided-attention requirements [29,30,43]. Parietal alpha suppression, although consistently associated with visuospatial attention, is also strongly influenced by visual complexity and sensory processing demands, making it sensitive to bottom-up perceptual load irrespective of attentional division [32,44]. Similarly, inverse frontal beta activity is significantly modulated by motor preparation, cognitive-motor interference, and acute stress responses, which limits its specificity as an attentional-split marker [34,35]. As a result, each neurometric captures only a partial aspect of the cognitive processes underlying divided attention. This limitation, increasingly discussed in the neuroergonomics and network-physiology literature, has led to the recognition that divided attention arises from the coordinated modulation of multiple neural systems, rather than from changes in a single oscillatory marker [18,23,24]. Converging evidence shows that attentional division engages distributed cortical networks spanning prefrontal, parietal and cingulate cortices, such that no individual frequency-band metric can fully capture the underlying neural dynamics [28,45]. Consequently, there is a growing need for multivariate methodologies capable of quantifying the shared neural information across neurometrics, approaches increasingly discussed in cognitive neuroscience [46] and neuroergonomics [19]. Such a framework forms the conceptual basis for the information-theoretic method introduced in the present study, which integrates multiple neurometric signals to capture their coordinated modulation during attentional splitting.

### 1.3. Rationale and objectives of the present study

The above-described state of the art led to increasing recognition, particularly within neuroergonomics and network physiology, that cognitive states such as attentional splitting emerge from distributed neural coordination rather than from modulations in one oscillatory band [19,46,47]. Building on this perspective, a number of recent studies have explored multivariate or information-theoretic approaches as tools to capture the complex interactions among neural signals. For example, entropy-based measures, transfer entropy, and mutual information (MI) have been applied to quantify nonlinear dependencies in EEG during workload changes, vigilance fluctuations, and multitasking behaviour [48–52]. These methods have been proven to be effective for analysing dynamic reconfigurations of brain networks, supporting the idea that multivariate metrics may capture cognitive processes more robustly than isolated spectral features.

Within this family of approaches, Mutual Information (MI) offers a particularly suitable mathematical framework for studying attentional allocation because it quantifies shared information among signals without assuming linearity or gaussian shape [53]. MI has therefore been used in EEG research primarily for feature selection, connectivity analysis [54], or classification tasks, for example in mental workload discrimination [55–59], driver state monitoring [52,60–64], and epilepsy detection [65–67]. Despite this, no study to date has leveraged MI to derive a single, interpretable, time-resolved index specifically aimed at quantifying attentional splitting. Existing MI applications typically operate on raw EEG channels or broad spectral features and do not integrate neurometrics that have been physiologically validated to capture different components of attentional allocation. Moreover, MI has not been used to explicitly track attentional splitting in real-world or ecologically realistic tasks such as driving.

This gap is particularly relevant in light of the evidence that attentional splitting depends on the joint modulation of multiple cortical networks, including frontal executive control, parietal attentional allocation, and sensorimotor coordination systems [28,45]. Characterizing this coordination requires a multivariate approach capable of capturing the interdependencies across neurometrics rather than treating each of them independently.

In response to this need, the present study introduces the Attentional Split Index (ASI), a Mutual-Information-based neurophysiological metric specifically designed to quantify the co-modulation of frontal theta, parietal alpha, and inverse frontal beta neurometrics during divided attention. By capturing shared information among these signals rather than their individual amplitudes, the ASI aims to isolate the specific neural signature of attentional splitting, overcoming confounds such as mental effort, visual complexity, or motor interference that affect traditional neurometrics. To evaluate its sensitivity and specificity, the ASI was tested in a simulated driving environment that manipulated attentional split across two contexts (Urban, Highway) and four task conditions (Focused, ACPT, Matrix, SURT). Based on the literature and the theoretical rationale above, we hypothesized that the ASI would: (1) increase systematically with the degree of attentional division; (2) show greater selectivity than univariate EEG neurometrics; and (3) exhibit strong convergence with both subjective and behavioural (eye-tracking) measures. The findings presented in this work provide the first demonstration of an MI-based neurometric specifically engineered to quantify attentional splitting in real-world operational scenarios.

## 2. Methods

### 2.1. Participants

Twenty-five healthy adult participants (18–45 years, mean age ± SD = 26 ± 6.3; 13 females), all licensed drivers with a minimum of three years of continuous driving experience (>10,000 km/year), were recruited for the study. Inclusion criteria were: normal or corrected-to-normal vision, no history of neurological or psychiatric disorders, no psychoactive medication usage, and no diagnosis of sleep disorders. Participants reported no prior familiarity with the specific cognitive tasks used in the experiment.

The sample size is consistent with previous neurophysiological studies on attentional allocation and divided attention in driving and dual-task paradigms (Lin et al., 2011; Ladouce et al., 2019; Hirano et al., 2020). All participants provided written informed consent. The research protocol was approved by the institutional ethics committee of PANACEA Project Consortium (Approval ID: 2022_07) and complied with the Declaration of Helsinki. The recruitment period started on 03rd May 2022 and ended on 24th June 2022.

### 2.2. Experimental design

Each participant completed eight driving segments organized in a 2 × 4 within-subject design, crossing:

- Driving context: Urban vs Highway
- Attentional-split condition:
  ◦ Focused driving (no secondary task)
  ◦ Auditory Continuous Performance Task (ACPT)
  ◦ Visual-cognitive Matrix task
  ◦ Visuo-manual SURT task

This structure mirrors validated paradigms for experimentally manipulating attentional division in operational settings [9,23,24]. Before the experimental session, participants received standardized instructions regarding both the primary driving task and the secondary tasks. They first completed an initial familiarization drive to become accustomed to the simulator and to the overall task structure. The experimenter also explained the response rules for each secondary task before data collection started.

#### 2.2.1. Primary driving task.

The experimental task was carried out in a high-fidelity driving simulator (Virtual Vehicle, Graz, Austria) equipped with a full vehicle mock-up mounted inside a VI-grade cave system. The system employed a 140° visual display generated by a three-projector setup arranged in a 3 × 4 k configuration, providing an immersive and continuous field of view of the road environment. Two auxiliary LCD panels positioned laterally served as virtual rear-view mirrors. Auditory immersion was achieved using a 5.1 surround-sound system (Creative, Singapore) reproducing engine, traffic, and environmental sounds, while four vibro-tactile transducers mounted beneath the vehicle chassis simulated engine vibrations and road surface irregularities, enhancing ecological realism. The driving scenarios used for the primary task, i.e., Urban and Highway, were developed in SCANeR Studio (v1.0, AVSimulation, France), enabling precise control over traffic density, road geometry, and environmental features. The Urban scenario was designed to reproduce a visually and operationally denser environment, characterized by a richer roadside scene and a higher need to distribute attention across multiple relevant sources of information. In contrast, the Highway scenario was designed to reproduce a more regular and less cluttered driving environment, with more stable road geometry and lower environmental fragmentation. The two contexts were therefore intended to differ primarily in ecological attentional demand rather than in the structure of the secondary tasks. The experimenter was positioned behind the participant, outside the participant’s field of view, and maintained constant communication through a two-way radio system to provide instructions and ensure safety throughout the experiment.

#### 2.2.2. Secondary tasks (manipulating attentional split).

Secondary-task stimuli were delivered through two dedicated channels depending on the task modality. Auditory distractors were presented via an independent stereo sound system positioned inside the cabin, while visual–manual distractors were administered through an Android tablet (Samsung, Seoul, South Korea) mounted to the right side of the dashboard to mimic an in-vehicle infotainment display. The tablet was remotely triggered by the experimenter, ensuring precise timing of secondary-task presentation without interfering with the primary driving task.

To progressively increase attentional division across modalities and response demands [15,68,69], the following secondary tasks were provided to the participants:

- Focused (no secondary task) – Drivers maintained lane and speed using full attention on the road.
- Auditory task (ACPT) – Participants monitored a continuous sequence of auditory stimuli and responded to infrequent target tones using a button press [70].
- Visual-cognitive Matrix task – Participants mentally solved 3D rotation matrices presented visually, responding via steering wheel buttons.
- Visuo-manual SURT task – Participants performed a standardised visual-manual tracking task with the steering wheel while driving, imposing the highest level of attentional division [71].

Each driving context (i.e., Urban or Highway) served as a “segment” within which the secondary tasks were embedded: within each context, each secondary-task condition was repeated twice in a random order, with each repetition lasting 60 s, followed by 20 s of undistracted “normal driving” (no secondary task, not relevant for the analysis). The relatively short duration of each block was intentionally selected to preserve the ecological continuity of the driving task and to avoid excessive fatigue, habituation, or strategic adaptation that may emerge in longer laboratory-style cognitive blocks. This choice is consistent with the neuroergonomic aim of the study, which was to probe attentional redistribution under realistic task transitions rather than to maximize the duration of isolated cognitive engagement. Moreover, each condition was repeated twice within each driving context, thus improving the reliability of the estimates. Importantly, participants drove continuously throughout each segment. The secondary tasks were not administered as separate phases replacing the driving task, but as superimposed demands embedded within an ongoing driving activity. The short intervals of undistracted driving between blocks were included to reduce carry-over effects and to restore a more natural driving state before the next attentional-split condition. A 120-second baseline preceded each segment. Additionally, 60-second eyes open (EO) and eyes closed (EC) conditions were collected at the beginning of the experimental session. The order of driving contexts and the ordering of secondary-task blocks were counterbalanced across participants to control for carry-over effects. Responses were provided through the interface associated with each task modality: button press for the auditory ACPT and steering-wheel-based responses for the Matrix task. Each secondary-task condition was repeated twice within each driving context.

**Fig 1:**
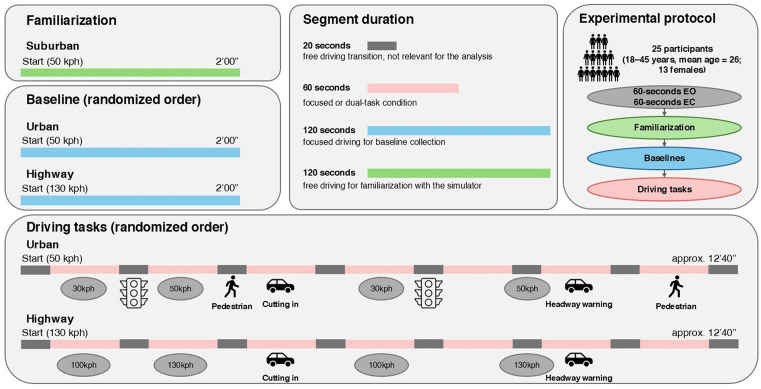
Graphical summary of the driving experiment structure. The protocol consisted of: (i) an initial familiarization drive, (ii) two baseline segments (Urban, Highway), and (iii) two task-drive segments, each containing randomized repetitions of four attentional-split conditions (Focused, ACPT, Matrix, SURT). Each secondary-task block lasted 60 s and was interleaved with 20 s of normal driving. Eyes-open (EO) and eyes-closed (EC) baselines were collected at the start of the session. The figure visually illustrates the temporal organisation, driving contexts, and randomization scheme.

A summary of the experimental protocol design is reported in Fig 1.

### 2.3. Subjective and behavioural data collection and analysis

After each driving segment, participants completed validated self-report scales assessing:

- Perceived attentional division
- Mental effort (5-point Likert scale)

The attentional-division rating was intended to capture the extent to which participants felt their attention had been distributed across concurrent demands during the preceding segment, whereas the mental-effort rating captured the perceived cognitive effort required by the same segment. Higher scores indicated greater perceived attentional division or effort. These instruments followed approaches used in prior EEG-driving research [14].

Additionally, eye-tracking measurements were collected along with the entire experimental sessions per each participant. More specifically, horizontal and vertical gaze, with their respective standard deviations, were considered.

### 2.4. Neurophysiological data collection and analysis

#### 2.4.1. EEG data acquisition and preprocessing.

EEG was recorded using the Mindtooth Touch EEG wearable device (Brain Products GmbH, Germany), which includes 8 wet (water-based) electrodes positioned according to the 10–20 system at AF7, AF8, AF3, AF4, P3, P4, Pz (sampling rate: 125 Hz). Reference and ground were placed on the mastoids. Impedances were maintained <100 kΩ [38]. The hardware and preprocessing methods follow validated protocols for operational neuroergonomics and real-world EEG monitoring [18,72].

EEG preprocessing followed established procedures compatible with mobile neuroergonomics:

- Band-pass filtering: 1–30 Hz (5th-order Butterworth)
- Artifact correction:
  ◦ Eye blinks removed using the o-CLEAN algorithm [73–75]
  ◦ Muscle/movement artifacts removed via joint probability and amplitude thresholds (±80 µV) via EEGLAB
- Rejection of segments with residual artifacts >20% of channel duration.

Individual Alpha Frequency (IAF) was computed from a 60-s eyes-closed baseline using the Gaussian fitting method described in [76].

#### 2.4.2. Neurometrics computation via global field power.

In the context of network physiology, a framework that examines how distributed neural and physiological processes dynamically cooperate to sustain functional states, the Global Field Power (GFP) provides a particularly valuable summary measure of large-scale cortical coordination. Rather than focusing on activity at individual electrodes, GFP captures the overall degree of spatial synchronization within a given frequency band, offering a compact yet sensitive descriptor of how neural assemblies reorganize during task engagement. This property has made GFP a useful tool in applied neurophysiological research, where it has been associated with variations in attentional allocation, sensory processing, and cognitive state.

Recent findings in network physiology further emphasize that the organization of neural oscillations is not static: patterns of synchrony and desynchrony, both within and across frequency bands, reconfigure systematically depending on behavioural demands and transitions between cognitive states [24,60,77]. These studies illustrate that large-scale coordination is an active mechanism supporting adaptation, rather than a by-product of local neuronal activity. In this perspective, GFP offers a principled way to quantify how attentional processes modulate the spatial structure of EEG rhythms, and therefore represents an appropriate foundation for extracting neurometrics relevant to attentional split.

In the present study, for each frequency band B(theta, alpha, beta), the GFP is computed as:


$$\begin{array}{c} GFP_{B}(t)=\sqrt{\frac{\text{1}}{N}{\left(x_{c,B}(t)-{\bar{x}}_{B}(t)\right)}^{\text{2}}} \end{array}$$ (1)

where:

- $x_{c,B}(t)$ is the band-limited EEG signal at channel c,
- ${\bar{x}}_{B}(t)$ is the spatial mean across channels,
- N is the number of electrodes.

Subsequently, the neurometrics extracted corresponded to:

- Frontal theta/beta ratio GFP (IAF-6 to IAF-2 Hz), linked to attentional demands [23,24].
- Parietal alpha GFP (IAF-2 to IAF + 2 Hz), which suppression reflects visual attentional engagement [14,78,79].
- Inverse frontal beta GFP (IAF + 2 to IAF + 16 Hz), reduced beta indicates attentional reallocation [24,80].

Frontal neurometrics were derived from the anterior electrodes AF7, AF8, AF3, and AF4, whereas the parietal neurometric was derived from the posterior electrodes P3, P4, and Pz. All neurometrics were computed using a 1-s sliding Hanning window (1 Hz resolution). This temporal windowing parameters were used for all frequency bands in order to preserve comparability across the neurometrics entering the ASI computation. Time series were then z-MAD normalised within driving context (i.e., Urban/Highway) following the procedure in [81] to allow within-subject comparisons across conditions.

### 2.5. ASI Computation via the Mutual Information Technique

The computation of the Attentional Split Index (ASI) was grounded in an information-theoretic framework designed to quantify the degree of coordinated modulation among multiple EEG neurometrics during task execution. The central rationale of this approach is that attentional division, unlike general increases in workload or arousal, emerges as a distributed neural phenomenon, characterised by simultaneous, interdependent changes across several cortical processes. Capturing this coordinated modulation therefore requires moving beyond univariate analyses and adopting a formalism capable of describing nonlinear statistical dependencies across time-varying neurometric signals. Mutual Information (MI) offers precisely this capability, as it quantifies the shared information between two variables without imposing assumptions of linearity, gaussian shape or monotonic coupling [82–84]. For these reasons, MI has been increasingly adopted in network physiology to examine temporal coordination among brain rhythms and to derive synthetic indices that reflect the integrated behaviour of multiple neural subsystems [49,54,67].

In the present study, MI was applied to the set of neurometrics derived through the GFP analysis, specifically frontal theta/beta ratio, parietal alpha, and inverse frontal beta activity, to construct a multivariate descriptor representing the degree of neural co-modulation associated with attentional splitting [85]. Prior research in network physiology has demonstrated that different neurometrics often exhibit characteristic patterns of coupling or decoupling depending on the behavioural state [52,54,57,86], and that these relationships can reorganize markedly when the brain transitions from focused attention to multi-stream monitoring, or from passive observation to active task engagement. The MI-based framework adopted here was therefore tailored to capture these reorganizations, under the hypothesis that situations requiring a division of attention would induce a synchronous modulation across neurometrics, reflected in an increase in shared information among them [87–89].

For each driving segment, the three neurometrics were first standardized and analysed within a sliding temporal window, allowing time-resolved estimation of MI. Given two neurometric time series X and Y, the pairwise MI was defined as:


$$\begin{array}{c} MI(X,Y)=\sum_{x\in X,y\in Y}p(x,y)\text{log}(\frac{p(x,y)}{p(x)p(y)})] \end{array}$$ (2)

where p(x) and p(y) are the marginal probability distributions of the two signals, and p(x,y) represents their joint distribution. Probabilities were estimated using a discretization procedure with equiprobable binning, a choice consistent with prior literature in operational neurophysiology and MI-based EEG analysis, as it ensures robust entropy estimation even for short windows or non-Gaussian distributions. This procedure yielded, for each time window, a 3×3 MI matrix:


$$\begin{array}{c} \text{M}(t)=[\begin{array}{ccc} \text{0} & MI\left(N_{\text{3}},N_{\text{1}}\right) & MI\left(N_{\text{3}},N_{\text{2}}\right)\\ MI\left(N_{\text{1}},N_{\text{3}}\right) & \text{0} & MI\left(N_{\text{1}},N_{\text{2}}\right)\\ MI\left(N_{\text{2}},N_{\text{3}}\right) & MI\left(N_{\text{2}},N_{\text{1}}\right) & \text{0} \end{array}] \end{array}$$ (3)

that encodes the full pairwise coupling structure among the neurometrics (i.e., $N_{X}$). The Attentional Split Index (ASI) for each window was then computed by aggregating the off-diagonal elements of this matrix:


$$\begin{array}{c} ASI(t)=\frac{\text{2}}{N(N-\text{1})}\sum_{i<j}MI\left(m_{i},m_{j}\right) \end{array}$$ (4)

with N=3 neurometrics, resulting in:


$$\begin{array}{c} ASI(t)=\frac{\text{1}}{\text{3}}\left[MI(\theta ,\alpha )+MI(\theta ,\beta )+MI(\alpha ,\beta )\right] \end{array}$$ (5)

This formulation provides a scalar quantity that increases when the neurometrics show coordinated fluctuations, interpreted as the neural signature of attentional division, and decreases when their temporal dynamics are more independent or driven by modality-specific processes. The windowed nature of the ASI further allows the temporal evolution of attentional splitting to be tracked continuously throughout the task, which is essential for studying real-world scenarios where cognitive demands fluctuate over time.

To ensure that increases in ASI reflected genuine neurophysiological coordination rather than spurious correlations or spectral leakage, a surrogate-data analysis was performed for each driving segment. To ensure that increases in ASI reflected genuine neurophysiological coordination rather than spurious correlations or spectral leakage, a surrogate-data analysis was performed for each driving segment. For every neurometric time series, we generated phase-randomized surrogates that preserve the original power spectrum (and thus the autocorrelation structure) while destroying any specific temporal alignment with the other neurometrics [90,91]. Concretely, for each surrogate realization, the time series was first transformed into the frequency domain using the Fast Fourier Transform (FFT). The amplitude spectrum was kept unchanged, whereas the phases were randomized by adding a set of independent random values uniformly distributed in [−π,π](with the constraint of Hermitian symmetry to obtain a real-valued signal). An inverse FFT (IFFT) was then applied to reconstruct a surrogate time series with identical amplitude distribution and autocorrelation as the original, but with phase structure, and hence temporal coordination, removed. For each driving segment, this procedure was repeated 1,000 times for one neurometric at a time (while leaving the others unchanged), and ASI was recomputed on each surrogate realization using the same sliding-window procedure as for the real data. This yielded, for every time window, a null distribution of ASI values under the hypothesis of no true inter-neurometric coupling. Real ASI values were then converted to z-scores relative to the surrogate distribution, providing a normalized measure of coordinated neurometric modulation that discounts the contribution of individual autocorrelation and power-spectral properties. This approach disrupts temporal coordination across signals but maintains their individual statistical characteristics, providing a conservative null model for MI estimation. For each segment, 1,000 surrogate ASI time series were generated, allowing a null distribution of ASI values to be constructed for every window.

Finally, the ASI values were correlated, through repeated measure correlation technique [92], with subjective ratings and behavioural indices to evaluate the convergent specificity and sensitivity of the index. This methodological framework enables the ASI to function as a principled and physiologically grounded biomarker of attentional splitting, capturing the temporal structure of neural coordination underlying the distribution of attention across concurrent task demands.

### 2.6. Statistical analyses

All statistical analyses were performed using MATLAB R2023a and R (version 4.3.1). The analytical pipeline was designed to evaluate the effects of Driving Context (Urban, Highway) and Attentional-Split Condition (Focused, Auditory, Matrix, SURT) across all classes of measurements: subjective reports, eye-tracking parameters, EEG neurometrics, and the ASI derived via MI. Unless otherwise specified, statistical significance was set at α = 0.05, and multiple comparisons were corrected using Bonferroni adjustments.

#### 2.6.1. Normalization and statistical tests.

Prior to inferential analysis, all subjective scores and neurometrics were z-normalized within participant and within driving context. For EEG-derived neurometrics, normalization was performed using z-MAD normalization relative to each context’s baseline to account for individual variability in absolute cortical activity. To assess the primary experimental effects, two-way repeated-measures ANOVAs (Context × Condition) were applied separately to each dependent variable:

- Subjective ratings (attentional division, mental effort)
- Eye-tracking parameters (horizontal gaze SD, fixation dispersion)
- EEG neurometrics (frontal theta/beta-GFP, parietal alpha-GFP, inverse frontal beta-GFP)
- ASI median values.

Greenhouse–Geisser correction was used where sphericity assumptions were violated. Significant interactions were explored through Bonferroni-corrected post-hoc pairwise comparisons. To characterize convergent specificity and sensitivity between measurement domains, the repeated measure correlation coefficients were calculated between:

- ASI and subjective attentional division.
- ASI and gaze-variability metrics.
- Eye-tracking and subjective measures.

False Discovery Rate (FDR) correction was applied across families of correlations. Correlation strength was interpreted according to Cohen’s conventions. All statistical tests were preceded by data normality of residuals (Shapiro–Wilk test), and homogeneity of variance (Levene test). Participants with >20% artifact-contaminated EEG data in more than one condition were excluded from neurophysiological analyses.

## 3. Results

The results are presented in five subsections: Subjective ratings, Neurophysiological Neurometrics, Mutual-Information-Based Attentional Split Index (ASI), Eye-Tracking Metrics, Correlation Analyses, and Surrogate Validation, in order to facilitate interpretability and correspondence with the Methods section.

### 3.1. Subjective ratings

Participants’ self-reported distraction showed a robust and systematic modulation across attentional-split conditions. A two-way repeated-measures ANOVA revealed a strong main effect of Condition (F = 39.568, p < .001, η²p ≈ 0.57), with distraction increasing monotonically from Focused to ACPT, Matrix, and SURT. More specifically, post-hoc analysis revealed that participants perceived the highest degree of distraction during the Matrix condition (all p < 0.02).

A smaller but significant main effect of Context emerged, with Urban driving eliciting higher perceived distraction than Highway driving, consistent with the increased environmental complexity. No significant interaction was observed.

These findings support the effectiveness of the task structure for eliciting progressive attentional-split demands.

### 3.2. Neurophysiological results: neurometrics

The three EEG neurometrics traditionally associated with attentional modulation, i.e., parietal alpha GFP, inverse frontal beta GFP, and the frontal theta/beta ratio, were analysed to assess their responsivity to increasing attentional-split demands.

**Fig 2:**
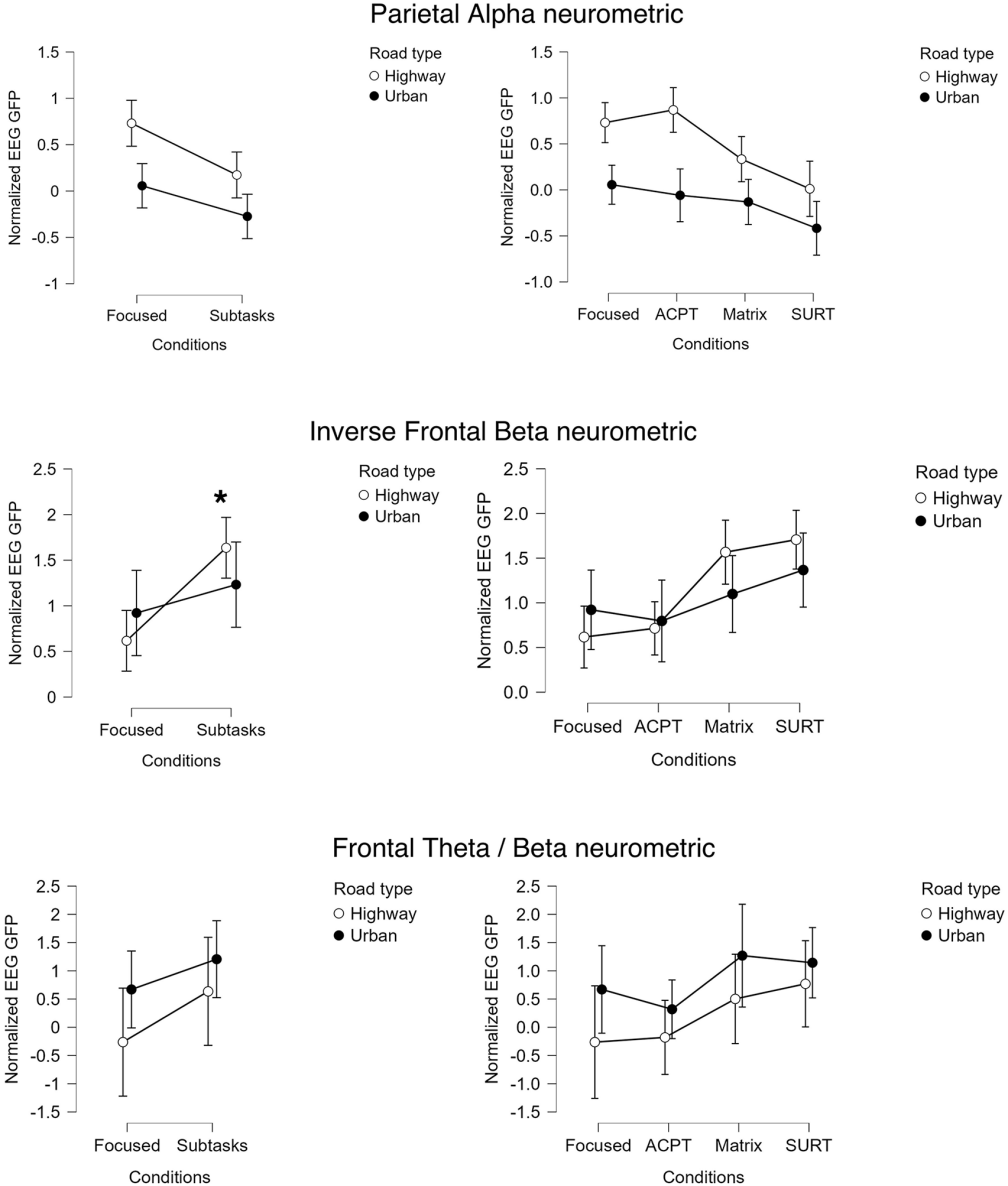
Group-level distributions of the three traditional neurometrics (i.e., Parietal Alpha GFP, Inverse Frontal Beta GFP, and Frontal Theta/ Beta ratio) computed in Urban and Highway contexts. Although Inverse Frontal Beta exhibited partial responsivity to task demands, none of the neurometrics selectively tracked attentional splitting. Parietal Alpha reflected visual complexity, while Frontal Theta/ Beta showed no systematic condition-related modulation.

Across participants, these neurometrics only partially exhibited significant Condition effects, replicating prior evidence that these markers are sensitive to cognitive and visual interference (Fig 2). In particular:

- Parietal alpha showed no significant main statistical effect across the experimental conditions (F = 2.697, *p* = 0.121, η²p ≈ 0.10).
- Inverse frontal beta increased progressively from Focused to SURT (ANOVA: F = 8.668, *p* = 0.01, η²p ≈ 0.27), reflecting greater cognitive engagement and motor interference. Post-hoc analysis revealed no significant differences between the Conditions and Contexts (i.e., Urban vs. Highway).
- The frontal theta/beta ratio also showed no significant main statistical effect across the experimental conditions (F = 1.881, *p* = 0.193, η²p ≈ 0.07).

Therefore, none of these neurometrics demonstrated a clear specificity to attentional splitting. These results reinforced the need for an alternative approach (i.e., neurometric) capable of isolating the *attentional-split* component.

### 3.3. Neurophysiological results: Mutual-Information-based Attentional Split Index (ASI)

The ASI showed a markedly different behavioural profile compared to the individual neurometrics, revealing a highly selective responsivity to attentional splitting.

A two-way repeated-measures ANOVA on ASI values revealed a significant main effect of Condition (F = 24.967, *p* < 0.001, η²p ≈ 0.51): ASI increased systematically from Focused and ACPT to Matrix and SURT, in both driving contexts. Additionally, post-hoc tests analyses revealed that the ASI resulted significantly higher during Matrix and SURT conditions, compared to the Focused and ACPT ones (all *p* < 0.01).

Unlike the traditional neurometrics, ASI also differentiated between Urban Focused and Highway Focused driving, indicating that the proposed index increased even without an explicit secondary task when the environment demanded more distributed attention (e.g., urban traffic, pedestrians, unpredictable events).

**Fig 3:**
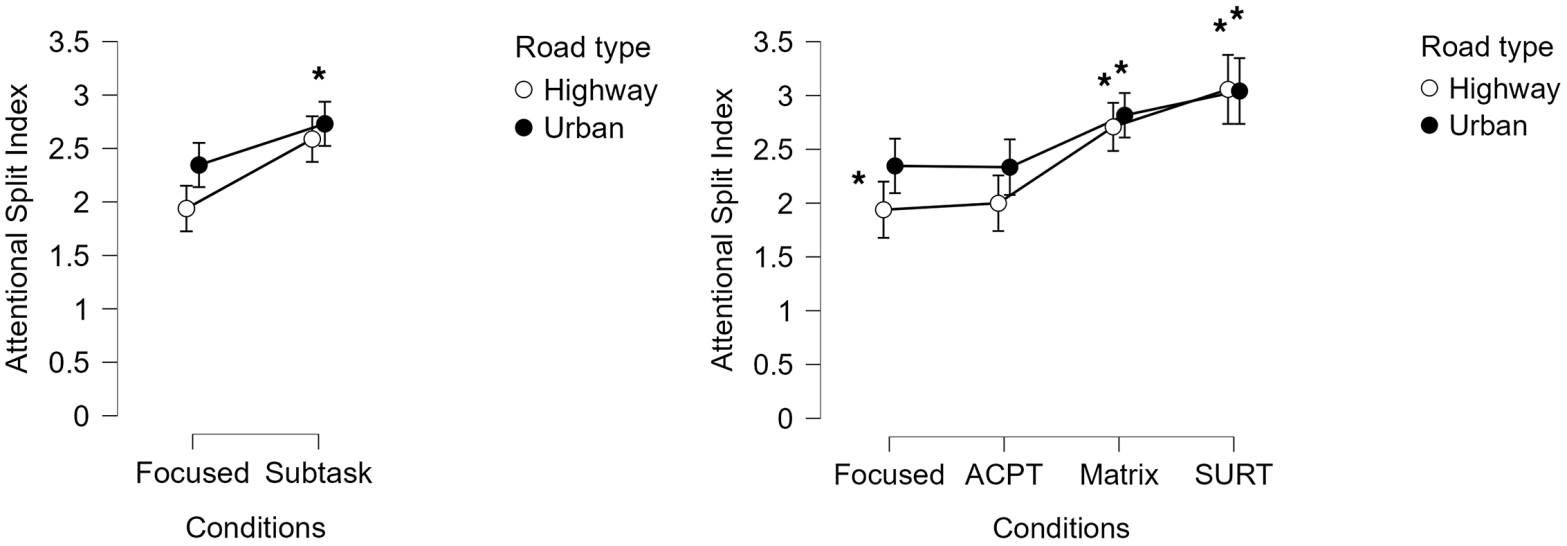
ASI values for the four attentional-split conditions in Urban and Highway environments. ASI exhibited a robust monotonic increase from Focused to SURT and successfully differentiated Urban from Highway even in the absence of explicit dual tasks. The index demonstrated the strongest sensitivity and specificity among all neurophysiological measures. Asterisks denote significant post-hoc contrasts.

The Context effect was present but smaller than the Condition effect (F = 6.967, *p* = 0.02, η²p ≈ 0.23), confirming that the index is driven predominantly by the degree of attentional division rather than general environmental complexity (Fig 3).

No significant Context × Condition interaction was observed, suggesting that the neural mechanism captured by ASI is consistent across driving environments. Overall, the ASI provided the clearest neurophysiological separation among the attentional-split conditions.

### 3.4. Eye-tracking results

**Fig 4:**
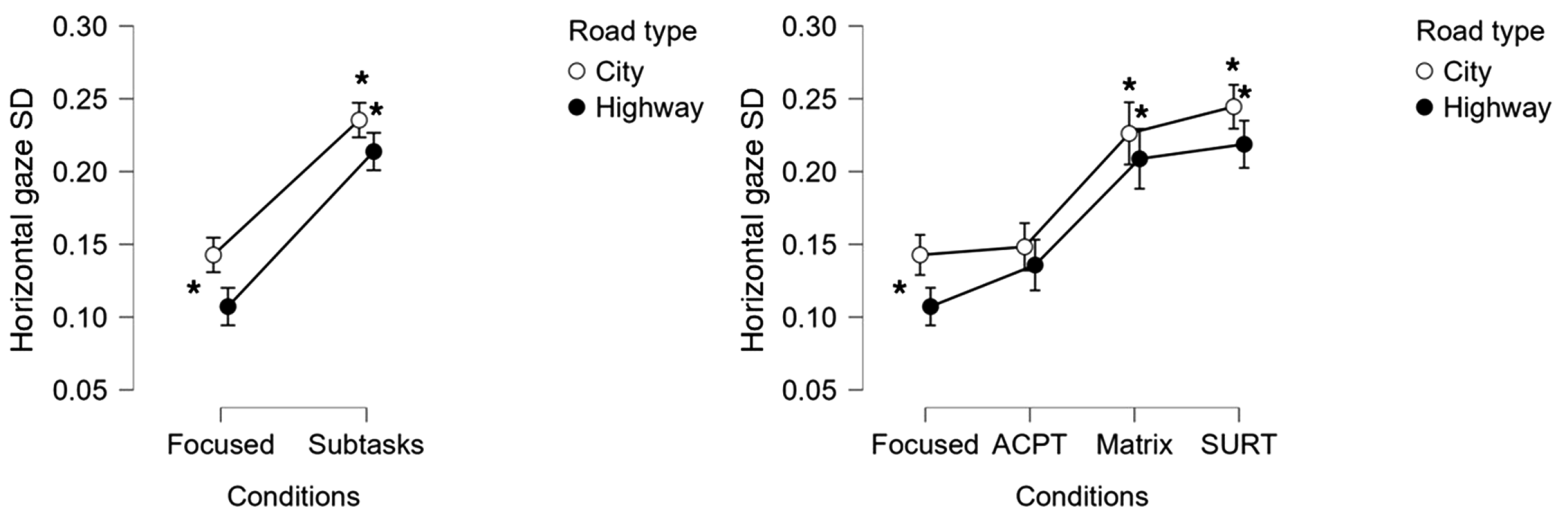
Horizontal gaze standard deviation (SD) for each condition and context. Gaze variability increased with attentional-split demands and was higher in the Urban environment, consistent with increased environmental complexity and attentional distribution. This pattern supports the behavioural sensitivity of the task hierarchy.

Horizontal gaze variability (gaze SD) showed a clear modulation pattern aligned with the attentional-split demands. A two-way repeated-measures ANOVA showed:

- Significant main effect of Condition (F = 44.989, *p* < 0.001, η²p ≈ 0.65), with SURT > Matrix > ACPT > Focused.
- Significant main effect of Context, with greater gaze dispersion in Urban than in Highway environments (F = 6.158, *p* = 0.02, η²p ≈ 0.20) (Fig 4).

These results are consistent with increased ASI across the visual scene when a secondary visual–manual task is present, and with previous findings linking gaze variability to attentional demands.

### 3.5. Correlation analyses

**Fig 5:**
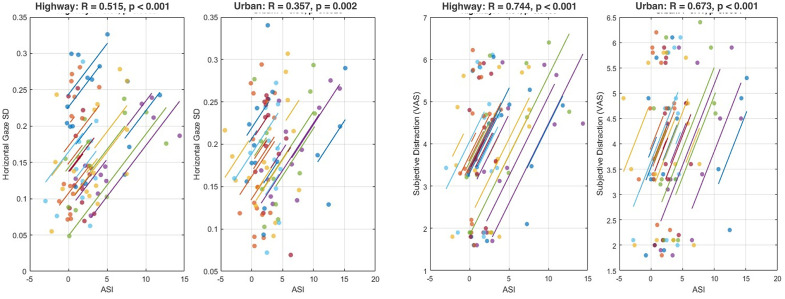
Scatterplots of repeated-measures correlations showing strong associations between ASI and (i) horizontal gaze variability and (ii) subjective distraction in both Urban and Highway contexts. The ASI exhibited substantially stronger correlations than traditional neurometrics (Table 1), supporting its convergent and ecological specificity and sensitivity as a neural measure of attentional splitting.

Repeated-measures correlation analyses were conducted to evaluate the convergent specificity and sensitivity of the ASI with both oculomotor behaviour and subjective distraction, computed separately for the Urban and Highway environments to account for context-specific attentional dynamics. Across both contexts, ASI showed significant and positive associations with horizontal gaze variability, indicating that stronger neural signatures of attentional split corresponded to broader fluctuations in gaze position. Specifically, ASI correlated with horizontal gaze SD with r = 0.515, p < .001 in the Urban environment and r = 0.357, p = .002 in the Highway environment (Fig 5, left). The larger effect observed in the Urban scenario is consistent with its inherently higher visual complexity, which requires more frequent shifts of attention across the scene and is therefore reflected in both neural and oculomotor indices.

ASI also demonstrated strong convergence with participants’ self-reported distraction. Repeated-measures correlations yielded r = 0.744, p < .001 in Urban driving and r = 0.673, p < .001 in Highway driving (Fig 5, right). These findings indicate that increases in shared-information dynamics among neurometrics, quantified by the ASI, closely parallel the participants’ introspective perception of attentional division across concurrent demands. Finally, a significant association was observed between horizontal gaze variability and subjective distraction (r = 0.48, p = .021), consistent with the interpretation that oculomotor dispersion reflects the perceived need to distribute attention across multiple stimuli. Notably, this correlation was weaker than those involving ASI, reinforcing the role of the ASI as a particularly sensitive and ecologically valid neurophysiological marker of attentional splitting.

In parallel, the same repeated measure correlation analysis was performed by considering the three EEG neurometrics associated with the attentional modulation. Table 1 shows how any of these neurometrics resulted significantly correlated with the subjective distraction perception and the ocular behaviour.

Collectively, these results confirm that the ASI captures a coherent, multimodal signature of attentional division that aligns strongly with both behavioural and subjective indicators across driving contexts. On the contrary, none of the univariate EEG features, i.e., parietal alpha, inverse frontal beta, or frontal theta/beta ratio, showed significant associations with subjective distraction or gaze variability in either driving context. These null results highlight a critical limitation of classical neurometrics: although they respond to specific cognitive or perceptual demands, they fail to covary systematically with the behavioural and introspective indicators of attentional-split dynamics.

**Table 1:** Summary of the repeated measure correlation analysis performed by considering the three EEG neurometrics associated with the attentional modulation and the subjective distraction reports and the ocular behaviour.

Driving environment	Parameters	Results
Urban	Parietal Alpha vs Subjective	R = −0.112, p = 0.271
	Parietal Alpha vs Gaze SD	R = − 0.098, p = 0.225
Highway	Parietal Alpha vs Subjective	R = −0.188, p = 0.118
	Parietal Alpha vs Gaze SD	R = −0.207, p = 0.139
Urban	Inverse Frontal Beta vs Subjective	R = 0.221, p = 0.089
	Inverse Frontal Beta vs Gaze SD	R = 0.191, p = 0.103
Highway	Inverse Frontal Beta vs Subjective	R = 0.271, p = 0.081
	Inverse Frontal Beta vs Gaze SD	R = 0.309, p = 0.069
Urban	Frontal Theta/ Beta vs Subjective	R = 0.091, p = 0.318
	Frontal Theta/ Beta vs Gaze SD	R = 0.106, p = 0.309
Highway	Frontal Theta/ Beta vs Subjective	R = 0.191, p = 0.115
	Frontal Theta/ Beta vs Gaze SD	R = 0.207, p = 0.102

### 3.6. Surrogate analyses

Surrogate data were used to assess whether ASI reflects genuine neurophysiological coordination or spurious temporal correlations.

Across all driving conditions, no surrogate ASI exhibited significant Condition or Context effects, confirming that the modulation observed in the real ASI is not attributable to statistical artefacts.

A final analysis quantified the percentage of participants for whom real ASI values significantly exceeded surrogate ASI values, yielding a clear pattern:

- SURT and Matrix showed the highest percentage of participants with real > surrogate ASI.
- ACPT showed intermediate percentages.
- Focused conditions showed lower but still non-random percentages.
- Eyes-open baseline (EO) showed by far the lowest percentage, close to chance.

The EO finding is crucial: because no attentional splitting occurs in this condition, ASI should not exceed surrogate values, and indeed, it does not. This result provides strong evidence that ASI is specifically responsive to the presence of attentional-split dynamics (Table 2).

**Table 2:** Summary of the significance percentage related to the ASI-real and ASI-surrogate comparison for each participant.

	Focused	ACPT	Matrix	SURT	EO
*Significance ASI-real vs. ASI-surrogate (%)*	74,1%	81,9%	92,6%	85,2%	11,1%

The following Table 1 resumes the main and significant outcome of the presented analysis in terms of neurophysiology-derived features for the attentional split characterization.

## 4. Discussion

### 4.1. Overview and objectives of the study

The primary objective of this work was to develop and provide an initial empirical assessment of the Attentional Split Index (ASI), a neurophysiological marker specifically designed to quantify how attention is distributed across concurrent task demands. As detailed in the Introduction, a major gap in the literature is the absence of a neural index that selectively captures attentional splitting, as opposed to more general constructs such as mental effort, visual complexity, sensory engagement, or motor interference. Existing EEG neurometrics, including frontal theta/beta ratio [29,30], parietal alpha suppression [32,14,79], and frontal beta desynchronization [24,34,36], have demonstrated responsivity to attentional demands but lack the sensitivity required to distinguish different degrees of divided attention due to different attentional demands. Furthermore, converging evidence from neuroergonomics and network neuroscience suggests that divided attention relies on distributed interactions across prefrontal, parietal, and cingulate networks rather than on isolated oscillatory mechanisms [19,28,45,46].

Motivated by this theoretical framework, the present study adopted a multivariate information-theoretic approach, grounded in Mutual Information, to capture the coordinated modulation among established neurometrics and to derive a selective, interpretable indicator of attentional splitting during ecologically realistic driving.

### 4.2. Traditional neurometrics: responsivity without specificity

The behaviour of classical neurometrics, i.e., parietal alpha suppression, inverse frontal beta, and the frontal theta/beta ratio, provides an important benchmark for interpreting the performance of the ASI. All three indicators exhibited modulation across experimental conditions, confirming their known responsivity to variations in visual input, cognitive engagement, or motor interference. These effects are consistent with past studies reporting increases in frontal theta under cognitive load, reductions in parietal alpha during visuospatial engagement, and frontal beta desynchronization during multitasking [18,23,24]. However, these neurometrics did not selectively track attentional splitting. Parietal alpha decreased not only during Matrix and SURT, but also during visually complex Urban driving, highlighting its dependence on visual load rather than attentional division per se. Similarly, inverse frontal beta reflected manual interference in SURT, and the frontal theta/beta ratio remained largely insensitive to the attentional-split hierarchy.

These findings reinforce a central point emerging in the literature and emphasized in the Introduction: univariate neurometrics conflate multiple cognitive dimensions and are therefore ill-suited for isolating divided attention. To provide a concise overview of the comparative behaviour of the EEG-derived measures, Table 3 summarizes the statistical outcomes for each neurometric and for the ASI. The pattern clearly illustrates the partial or absent sensitivity of traditional neurometrics to attentional-split demands, and the markedly superior specificity shown by the ASI

**Table 3:** Comparative overview of the statistical effects observed for Parietal Alpha, Inverse Frontal Beta, Frontal Theta/Beta ratio, and the MI-based ASI. The table reports the presence of significant Context and Condition effects, interactions, main ordering across conditions, and a qualitative interpretation of each measure’s sensitivity and specificity to attentional splitting. ASI shows the strongest and most selective modulation.

Measure	Context effect	Condition effect	Interaction	Main ordering	Key interpretation
Parietal Alpha	No	No	No	SURT>Matrix>ACPT>Focused	Non-specific and non-sensitive.
Inverse Frontal Beta	No	Partial	No	SURT>Matrix>ACPT>Focused	Partly Sensitive but not modality-driven.
Frontal Theta/ Beta	No	No	No	SURT>Matrix>ACPT>Focused	Non-specific and non-sensitive.
ASI	✓	✓✓ (strong)	No	SURT>Matrix>ACPT>Focused	Specific to attentional split.

### 4.3. The ASI as a multivariate neural signature of attentional splitting

In contrast to the behaviour of traditional neurometrics, the Attentional Split Index (ASI) demonstrated a clear, systematic, and theoretically coherent sensitivity to the degree of attentional division imposed by the experimental conditions. Across both Urban and Highway contexts, ASI values increased monotonically from Focused to ACPT, Matrix, and SURT, closely mirroring the hierarchy of attentional demands defined a priori. This pattern highlights an aspect that EEG research has repeatedly emphasised: divided attention does not manifest through the modulation of a single oscillatory component, but through the coordinated reconfiguration of distributed cortical systems [28,45,50]. The ASI capitalises on this principle by quantifying the shared information among neurometrics (i.e., frontal theta/beta ratio, parietal alpha, and inverse frontal beta) that represent distinct physiological mechanisms of attentional control, sensory processing, and cognitive stability [32,34,43].

A particularly relevant finding is that the ASI detected attentional-split dynamics even in the absence of explicit secondary tasks. During Focused driving, ASI values were significantly higher in the Urban context than in the Highway context, despite neurometric amplitudes showing no comparable specificity. This result aligns with evidence that environmental complexity modulates attentional distribution by increasing the need for rapid shifts between stimuli and internal processing demands [9]. Crucially, because the ASI indexes the covariation of neurometrics rather than their absolute levels, it appears sensitive to subtle redistributions of attentional resources that go unnoticed when each neurometric is analysed in isolation. This behaviour is consistent with the broader framework of network neuroscience, according to which cognitive states, including divided attention, are best characterised by the integration and coordination of multiple neural processes rather than isolated regional activity [89].

The ASI’s performance also reflects the advantages of using Mutual Information (MI) as the computational core. Unlike correlation-based or linear-feature-combination approaches, MI captures nonlinear relationships among neurometrics and does not rely on Gaussian assumptions [82]. Previous work has shown that MI is particularly well suited to detect subtle, dynamic couplings among EEG features during changes in workload, vigilance, or multitasking [49,50,52]. However, no prior study has applied MI to derive a single, physiologically interpretable index explicitly targeting attentional splitting. The present findings show that MI can successfully integrate complementary neurometric signatures into a cohesive, state-specific measure that selectively responds to the phenomenon of interest.

Overall, the ASI provides the first evidence that co-modulation across attentional neurometrics represents a robust and selective neural signature of attentional splitting. Whereas traditional neurometrics confound attentional division with effort, visual load, or motor activity, the ASI reliably isolated the neural pattern corresponding to the redistribution of attentional resources across multiple concurrent demands. This multivariate, information-theoretic approach therefore represents a methodological advance aligned with current theoretical models of distributed attention and provides a solid foundation for future neuroergonomic applications requiring real-time monitoring of attentional allocation.

### 4.4. Subjective and behavioural validation of attentional split

Consistent with the experimental manipulation, subjective distraction increased monotonically from Focused to SURT, replicating classical dual-task and multitasking findings [4,5]. Importantly, participants also reported greater distraction during Urban compared to Highway driving, confirming that the operational context independently modulates attentional distribution. These subjective results serve as a reference framework for assessing the ecological coherence of the ASI, indicating that the task hierarchy effectively elicited different degrees of attentional division.

Horizontal gaze variability increased significantly across attentional-split conditions and was higher in Urban driving, consistent with well-established evidence that gaze dispersion reflects attentional distribution and environmental scanning [5]. The observed pattern supports the behavioural relevance of the task hierarchy.

The strong repeated-measures correlations between ASI and gaze variability (Urban: r = 0.515; Highway: r = 0.357) reinforce the interpretation that ASI captures neurophysiological processes that are behaviourally expressed through the oculomotor system. The stronger Urban correlation reflects the higher attentional fragmentation and environmental monitoring needs in that setting. Similarly, the ASI showed remarkably strong associations with subjective distraction in both driving environments (Urban: r = 0.744; Highway: r = 0.673). These are large effect sizes in the cognitive neuroscience domain and suggest that ASI captures cognitive processes directly aligned with individuals’ moment-to-moment experience of attentional splitting. This strong neural–phenomenological correspondence provides clear evidence that ASI is not merely sensitive to task-induced artefacts or generic cognitive load, but tracks the perceived distribution of attention. This complements the behavioural correlations and strengthens the ecological coherence of the ASI. Furthermore, the correlation between gaze variability and subjective distraction (r = 0.48) supports the common interpretation that gaze dispersion is a behavioural marker of attentional allocation, although it is less sensitive than ASI.

Worth noting is the fact that, despite SURT representing the condition with the highest level of attentional division, participants reported higher subjective distraction during the Matrix condition. At first glance, this appears counterintuitive, given that SURT imposes the strongest visuo–manual dual-task interference. However, this pattern can be explained by considering that the Matrix task required a substantially higher mental effort than SURT. To investigate this possibility, we computed an additional neurometric of mental effort, defined as the GFP in the theta band extracted from frontal electrodes, in line with the literature linking frontal-theta increases to cognitive effort and working-memory demands [93,94]. This analysis revealed that frontal theta-GFP was significantly higher in the Matrix condition compared to SURT, confirming that the Matrix task imposed a stronger cognitive load despite requiring less manual interaction. This finding provides a coherent explanation for the subjective ratings: although attentional splitting is higher in SURT, the subjective feeling of distraction is likely dominated by the mental effort required to perform the Matrix task. In other words, participants may experience Matrix as more cognitively intrusive, even if, from a neurophysiological point of view, the degree of attentional splitting (as captured by the ASI) is lower than in SURT. This distinction reinforces the importance of the ASI: unlike subjective ratings, which can conflate attentional splitting with cognitive effort, the ASI isolates the specific neural signature of divided attention, independently of the mental workload associated with the secondary task.

### 4.5. Surrogate analyses confirm the specificity of ASI

The surrogate-data analysis provides strong evidence that ASI modulations reflect genuine neural coordination rather than statistical artefacts or covariations driven by overall EEG amplitude. When neurometric time series were phase-randomized, thereby preserving their power spectra while destroying temporal interdependencies, all experimental effects disappeared. ASI values in the surrogate data were indistinguishable across Conditions and Contexts, and the percentage of participants for whom real ASI exceeded surrogate ASI dropped sharply in the eyes-open baseline. This pattern demonstrates that the ASI is specifically driven by task-dependent neural co-modulation and is not inflated by autocorrelation, overall spectral power, or spurious coupling, addressing, therefore, a known limitation in EEG multivariate and connectivity metrics [48,95]. Together, these findings confirm that the ASI selectively indexes the neural mechanisms associated with divided attention.

### 4.6. Limitations and future directions

Although promising, the present study has some limitations. First, the number of EEG channels was limited, as inherent in wearable operational systems, and future work may explore whether denser montages improve ASI resolution. Second, while the ASI captures attentional splitting with high specificity, future research should examine its behaviour in other operational contexts (e.g., aviation, surgical environments) where routine activities imply multitasking situations. In this regard, combining ASI with complementary physiological modalities (e.g., pupillometry, fNIRS) may enhance multimodal estimation of attentional states. Thirdly, and probably most importantly, the present study does not constitute a formal predictive validation of the ASI. The evidence provided here demonstrates sensitivity, specificity within the tested paradigm, behavioural and subjective coherence, and robustness against surrogate-data controls, but it does not yet establish classification performance on unseen data or generalization across independent datasets. A stronger validation framework will therefore require future studies implementing subject-wise cross-validation, independent test sets, and threshold-based or classifier-based evaluations of ASI performance in real-world applications... The present study, contextualized in a real use case, has to be intended as a preliminary tool paving the way for new forefront approaches in monitoring and enhancing human performance and safety. Moreover, the use of a common 1-s analysis window across frequency bands ensured temporal consistency across neurometrics but implies different spectral sampling conditions for theta, alpha, and beta rhythms. In particular, lower-frequency activity is represented by fewer cycles within each window, which may reduce sensitivity to finer theta fluctuations. Future studies may therefore evaluate whether band-specific or adaptive window lengths improve the stability and interpretability of the ASI. Finally, because the ASI is a composite index obtained by aggregating pairwise mutual-information terms among multiple neurometrics, its interpretability at the level of single component contributions is necessarily reduced.

Future studies should therefore examine the behaviour of the individual MI pairs to better disentangle which inter-neurometric relationships drive the index under different attentional conditions and, additionally, should investigate whether ASI can be integrated into adaptive interfaces that dynamically respond to the user’s real-time attentional allocation.

## 5. Conclusion

This study introduced and provided initial empirical support for the Attentional Split Index (ASI), a Mutual-Information-based EEG metric specifically designed to quantify how attention is distributed across concurrent task demands in ecologically realistic scenarios. Addressing a long-standing gap in applied neuroscience, the ASI isolates the neural signature of attentional splitting by capturing coordinated fluctuations across multiple neurometrics, overcoming the non-specificity of traditional univariate EEG markers.

Across driving contexts and task conditions, the ASI showed a clear and systematic increase with the degree of attentional division, successfully distinguishing explicit dual-task demands as well as more subtle environmental influences such as the increased visual and cognitive complexity of Urban driving. Its strong correlations with both horizontal gaze variability and subjective distraction ratings confirm the ASI’s ecological coherence and demonstrate tight correspondence between neural, behavioural, and phenomenological indicators of attentional allocation. Surrogate-data analyses further established that the ASI reflects genuine neural coordination rather than artefactual coupling, providing robust evidence of its specificity to attentional-split dynamics.

Taken together, these findings position the ASI as a novel and practical tool for neuroergonomics, offering a principled basis for real-time monitoring of attentional allocation in complex operational environments. By leveraging multivariate information-theoretic principles, the ASI represents a step forward toward next-generation human–machine systems capable of adapting to the user’s cognitive state. Future work will pursue formal predictive validation of the ASI across additional domains, including further automotive scenarios and other operational contexts in which the attentional split processes monitoring are crucial, such as the learning-related ones [96], and will explore its integration into adaptive closed-loop interfaces.
